# Genome-wide methylomic analysis in individuals with *HNF1B* intragenic mutation and 17q12 microdeletion

**DOI:** 10.1186/s13148-018-0530-z

**Published:** 2018-07-18

**Authors:** Rhian L. Clissold, Beth Ashfield, Joe Burrage, Eilis Hannon, Coralie Bingham, Jonathan Mill, Andrew Hattersley, Emma L. Dempster

**Affiliations:** 1 University of Exeter Medical School, University of Exeter, Exeter, UK; 2 Exeter Kidney Unit, Royal Devon and Exeter NHS Foundation Trust, Exeter, UK

**Keywords:** Epigenetics, DNA methylation, HNF1B, CNV, 17q12 deletion, Renal disease, Autism

## Abstract

Heterozygous mutation of the transcription factor *HNF1B* is the most common cause of monogenetic developmental renal disease. Disease-associated mutations fall into two categories: *HNF1B* intragenic mutations and a 1.3 Mb deletion at chromosome 17q12. An increase in neurodevelopmental disorders has been observed in individuals harbouring the 17q12 deletion but not in patients with *HNF1B* coding mutations.

Previous investigations have concentrated on identifying a genetic cause for the increase in behavioural problems seen in 17q12 deletion carriers. We have taken the alternative approach of investigating the DNA methylation profile of these two *HNF1B* genotype groups along with controls matched for age, gender and diabetes status using the Illumina 450K DNA methylation array (total sample *n* = 60).

We identified a number of differentially methylated probes (DMPs) that were associated with HNF1B-associated disease and passed our stringent experiment-wide significance threshold. These associations were largely driven by the deletion patients and the majority of the significant probes mapped to the 17q12 deletion locus. The observed changes in DNA methylation at this locus were not randomly dispersed and occurred in clusters, suggesting a regulatory mechanism reacting to haploinsufficiency across the entire deleted region.

Along with these deletion-specific changes in DNA methylation, we also identified a shared DNA methylation signature in both mutation and deletion patient groups indicating that haploinsufficiency of *HNF1B* impacts on the methylome of a number of genes, giving further insight to the role of HNF1B.

**Electronic supplementary material:**

The online version of this article (10.1186/s13148-018-0530-z) contains supplementary material, which is available to authorized users.

## Introduction

The most common monogenic cause of developmental kidney disease is heterozygous mutation of the hepatocyte nuclear factor 1β (*HNF1B*) gene, located at chromosome 17q12 [1–3]. This gene encodes a transcription factor with important roles in the development of the kidney, pancreas, genital tract and liver [4]. Renal cysts are the most frequently observed clinical feature in HNF1B-associated renal disease, although the phenotype is very variable despite the single genetic aetiology [5]. Abnormalities are often detected on prenatal ultrasonography, where bilateral hyperechogenic kidneys with normal or slightly increased size are commonly found [6]. The prevalence of HNF1B-associated renal disease depends on which study cohort is selected; the detection rate ranges from 5% in children ≤ 16 years with renal aplasia/hypoplasia and chronic kidney disease to 31% in children with renal cysts, hyperechogenicity, hypoplasia or a single kidney [2, 7].

Extra-renal phenotypes are also common and include early-onset diabetes mellitus, pancreatic hypoplasia, genital tract malformations and abnormal liver function tests [8–13]. The mean age at diagnosis of diabetes is 24 years but can vary from the neonatal period to late middle age [14]. The pathophysiology reflects a combination of β cell dysfunction and insulin resistance; dysfunction of β cells results in reduced insulin secretion and is likely to be a consequence of pancreatic hypoplasia [10]. Most patients require treatment with insulin [14].

Heterozygous mutations in *HNF1B* generally occur in two forms; approximately 50% of patients are affected by an intragenic mutation (base substitution or small insertion/deletion within the *HNF1B* gene) with the other common mutation being a ~ 1.3 Mb deletion at chromosome 17q12, which encompasses the entire *HNF1B* gene [15, 16]. This region of chromosome 17 is susceptible to genomic rearrangement, which is mediated by non-allelic homologous recombination between flanking segmental duplications [17]. Interestingly, the recurrent 17q12 deletion has also been associated with neurodevelopmental disorders, such as autism spectrum disorder and attention-deficit hyperactivity disorder [18, 19]. The 1.3 Mb deleted region contains 14 genes in addition to *HNF1B*, and it is not currently clear what genetic mechanism gives rise to this neurodevelopmental comorbidity. Until recently, there was no evidence to suggest a genotype-phenotype correlation for any of the other clinical features seen in HNF1B-associated disease; this is consistent with haploinsufficiency as the underlying disease mechanism [15, 16]. However, recent work by Dubois-Laforgue and colleagues has shown renal function may be worse in patients with an *HNF1B* intragenic mutation than in those with a deletion [20].

Expression of the phenotype can vary significantly between families carrying the same *HNF1B* mutation and even between affected members of the same family, which suggests that additional genetic and/or environmental modifiers might influence the *HNF1B* phenotype. Most efforts to understand this phenotypic variation have concentrated on studying changes in the DNA sequence. To date, epigenetic mechanisms which act to developmentally regulate gene expression via modifications to DNA, histone proteins and chromatin have not been explored. DNA methylation is the most well characterised and stable epigenetic modification, influencing gene expression via the disruption of transcription factor binding and the attraction of methyl-binding proteins that initiate chromatin compaction and gene silencing. DNA methylation can be directly affected by DNA sequence variation—including large structural variants—both locally (*in cis*) and at more distal locations in the genome (*in trans*) [21–24]. In this study, we set out to characterise the epigenetic signature of HNF1B-associated disease and to determine if the signature differs depending on mutation classification: we profiled genome-wide patterns of DNA methylation in 20 individuals with an *HNF1B* intragenic mutation (HNF1Bmtn), 20 individuals with a 17q12 deletion encompassing *HNF1B* (17q12del) and 20 controls (ctrl) matched for age, gender and diabetes status.

## Methods

### Sample description

DNA was isolated from whole blood collected from unrelated individuals with HNF1B-associated disease who had been referred for genetic testing to Exeter Molecular Genetics Laboratory from 1998 to 2012; the criterion for initial referral was suspicion of HNF1B-associated disease by the referring clinician. DNA was isolated using standard phenol:chloroform methods and checked for quality and purity. Informed consent was obtained to perform *HNF1B* genetic testing as part of their clinical care, and the study was conducted in agreement with the Declaration of Helsinki Principles. Mutation screening was performed by sequencing of coding exons and exon-intron boundaries together with gene dosage assessment by multiplex ligation-dependent probe amplification (MLPA) as previously described [16, 25]. Control DNA was collected from two sources: (i) for individuals without diabetes mellitus, controls were individuals referred to Exeter Molecular Genetics Laboratory as above but with no *HNF1B* gene mutation or deletion detected on genetic testing; (ii) for individuals with diabetes mellitus, controls were individuals from the UNITED (Using pharmacogeNetics to Improve Treatment in Early-onset Diabetes) study with presumed type 1 diabetes mellitus based on a urine C-peptide/creatinine ratio ≥ 0.2 nmol/mmol with positive islet autoantibodies [26]. All individuals had a serum creatinine level < 250 μmol/L and were matched for age, sex and presence of diabetes (Table 1). All samples included in the study were unrelated. Two patients in the 17q12 deletion group had known neurodevelopmental disease (a 10-year-old female with developmental delay and a 31-year-old male with Asperger’s syndrome).

**Table 1:** Sample demographic table

	*HNF1B* intragenic mutation (*n* = 21)	17q12 deletion (*n* = 21)	Control* (*n* = 21)
Median age, years (interquartile range)	10 (2–28)	9 (2–31)	9 (2–29)
Sex, *n* (%)	Male 8 (38)	Male 8 (38)	Male 8 (38)
Diabetes, *n* (%)	9 (43)	9 (43)	9 (43)
Renal abnormality, *n* (%)			
-Renal cysts/cystic dysplasia	12 (57)	12 (57)	8 (38)
-Renal hyperechogenicity	2 (10)	1 (5)	2 (10)
-Single kidney	1 (5)	1 (5)	1 (5)
-Multicystic and dysplastic kidney	2 (10)	2 (10)	1 (5)
-Obstruction	–	2 (10)	–

The samples were matched for age, sex and diabetes status; all individuals had a serum creatinine level < 250 μmol/L

*For individuals without diabetes mellitus, controls were individuals with no *HNF1B* gene mutation or deletion detected on genetic testing; for individuals with diabetes mellitus, controls were individuals from the UNITED (Using pharmacogeNetics to Improve Treatment in Early-onset Diabetes) study with presumed type 1 diabetes

### Methylomic profiling

Blood-derived DNA was profiled using the HumanMethylation450 BeadChip (Illumina, San Diego, CA, USA) and scanned on an Illumina HiScan System (Illumina, San Diego, CA, USA). Illumina Genome Studio software was used to extract the raw signal intensities of each probe (without background correction or normalisation). Signal intensities for each probe were imported into R [27] using the *methylumi* and *minfi* packages [28, 29]. Multidimensional scaling plots of sex chromosome probes were used to check that the predicted sex corresponded with the reported sex for each individual. The 65 SNP probes, cross-hybridising probes [30, 31] and probes containing a SNP 10 bp from the extension position (MAF > 0.05) were excluded from analysis [30]. The ‘pfilter’ function of the *wateRmelon* package [32] was used to filter data by beadcount and detection *P* value. Samples with > 1% probes with a detection *P*value > 0.01 were removed, along with probes with a detection *P* value > 0.05 in at least 1% of samples and/or a beadcount < 3 in 5% of samples were also removed. The ‘dasen’ function in *wateRmelon* was used to normalise the data as previously described [32]. The number of samples that passed quality control was 60 (*HNF1B* intragenic mutation (*n* = 20), 17q12 deletion (*n* = 20) and control (*n* = 20) and a total of 388,295 CpG sites were included in the final dataset.

### Bisulfite pyrosequencing

A selected region was chosen to verify the array findings using the complementary technology of bisulfite pyrosequencing using the Qiagen Q24 Pyrosequencer (Qiagen, Hilden, Germany) (see [33]). Primers, probes and the PCR conditions for the selected region can be found in Additional file 1: Table S1; the sequencing reaction was performed using the manufacturer’s protocol.

### Statistical analysis

#### Differentially methylated position (DMP)

The one-way analysis of variance (ANOVA) test was used to test for differentially methylated sites associated with one of the three groups: *HNF1B* intragenic mutation (*n* = 20), 17q12 deletion (*n* = 20) or control (*n* = 20). DNA methylation values for each probe were regressed against *HNF1B* status with covariates for age, gender, diabetes status and cellular composition. As cell count data were not available for these DNA samples these were estimated from the DNA methylation data using both the Epigenetic Clock software [34] and the Houseman algorithm [35, 36]. To determine which group was driving the association behind the significant ANOVA results, the *T* statistics for controls versus each of the two *HNF1B* distinct genetic groups were extracted from the regression model. The presence of the 17q12 deletion was confirmed by analysing the DNA methylation raw data using the function ‘champ.CNA’ from the package ChAMP [37].

#### Differentially methylated regions (DMRs)

The *P* values for the comparisons between the control group and *HNF1B* genotype status from the regression analysis were converted into BED files and run through the *comb-p* [38] pipeline with a seed of (5 × 10^−4^) and distance parameter set to 500 bp. Briefly, *comb-p* generates DMRs by (1) calculating the auto-correlation between probes to adjust the input DMP *P* values using the Stouffer-Liptak-Kechris correction, (2) running a peak finding algorithm over these adjusted *P* values to identify enriched regions around a seed signal, 3) calculating region *P* value using the Stouffer-Liptak correction and (4) correcting for multiple testing with the one-step Šidák correction. Significant regions were identified as those with at least two probes and corrected *P* value < 0.05.

## Results

**Fig. 1:**
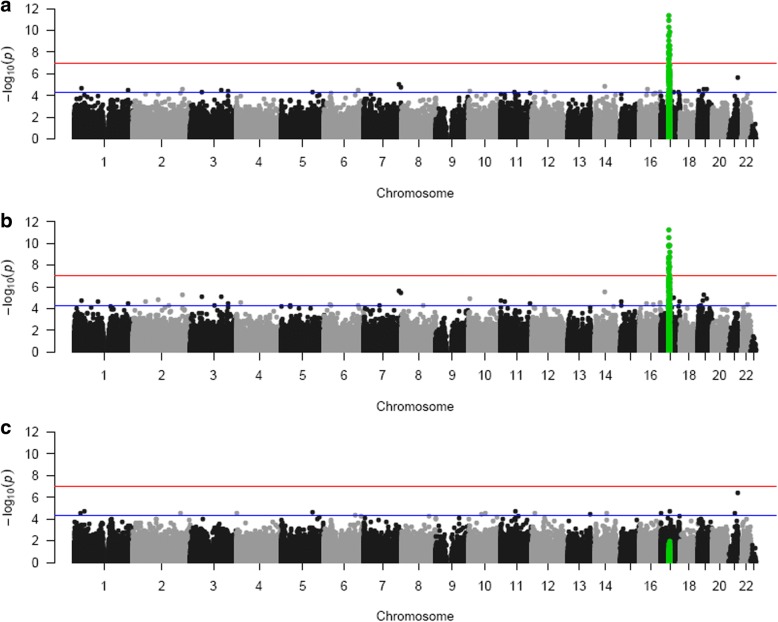
Manhattan plots of the three analyses. **a** *P* values from the ANOVA test on the three genotype groups (HNF1B intergenic mutation, 17q12 deletion and controls). Highlighted in green are the 450K probes that are located in the 17q12 deletion region. **b** *P* values from the *T* statistic from the regression model on controls compared to the 17q12 deletion samples. **c** *P* values from the *T* statistic from the regression model on controls compared to the *HNF1B* mutation samples. (The red line indicates experiment-wide significance threshold (*P* < 1 × 10^−7^), and the blue line is a more relaxed ‘discovery’ threshold of *P* < 5 × 10^–5^)

**Fig. 2:**
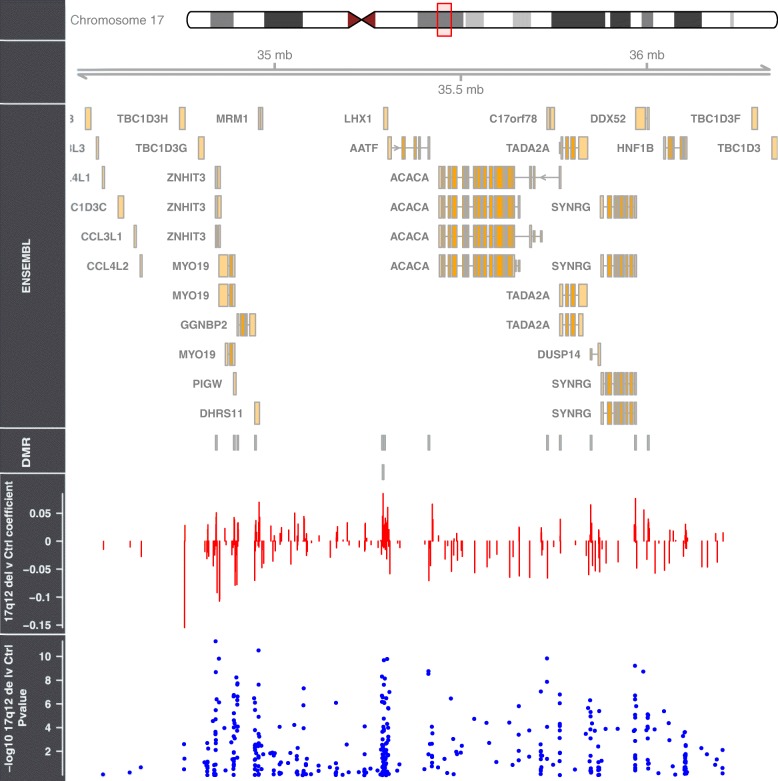
This figure illustrates the 17q12 deletion locus with the identified DMRs highlighted across the region. The regression coefficient and *P* values from the analysis comparing the 17q12 deletion subject with controls illustrates that both hyper and hypo DNA methylation changes are associated with the deletion

**Fig. 3:**
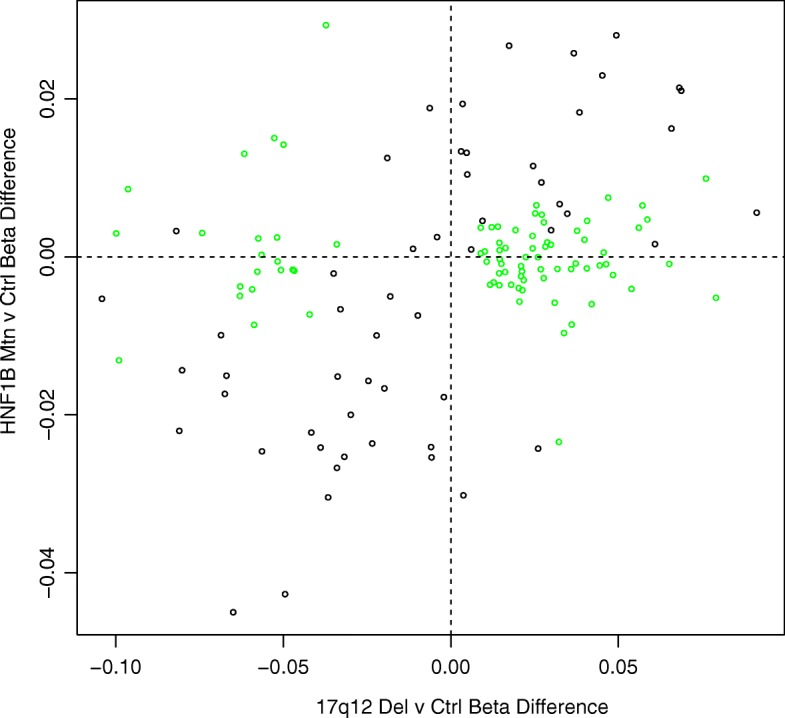
This scatterplot demonstrates the concordance in effect size between the two *HNF1B* genetic groups compared to controls when probes in the 17q12 deletion region (coloured green) are discounted

All samples characterised as 17q12 deletion carriers were confirmed by the CNV calling function in CHAMP which uses the intensity data from the DNA methylation array (see Additional file 2: Figure S1). The probe-wise ANOVA analysis on the three groups *(HNF1B* intragenic mutation, 17q12 deletion and control) identified 21 differentially methylated positions (DMPs) (see Table 2) that passed our experiment-wide significance threshold (*P* < 1 × 10^−7^), representing a 5% family-wise error-rate estimated from 5000 permutations (see [39]), with 94 additional DMPs reaching a more relaxed ‘discovery’ threshold of *P* < 5 × 10^−5^ (Additional file 3: Table S2). To determine which group was driving this association, we extracted the *P* values for the *T* statistics from the model *HNF1B* intragenic mutation vs control and 17q12 deletion vs control; these results clearly show that the 17q12 deletion group is driving most of the associations seen in the ANOVA (see Table 2). All of the significant DMPs (*P* < 1 × 10^−7^) are located in the 17q12 deletion region (see Figs. 1 and 2) suggesting that the deletion is exerting effects in cis. While this region appears to be driving the main associations observed, probes situated outside the deletion region, which are associated with both *HNF1B* genotype groups, are strongly correlated (*r* = 0.63, *P* = 2.626E−07); see Fig. 3 and Additional file 4: Table S3 for a list of probes that are significantly differently methylated in both *HNF1B* genotype groups compared to controls.

**Table 2:** Top results from the probe-wise ANOVA analysis. The T statistic results from the regression analysis indicate that the association is mainly driven by the 17q12 deletion patients. DMP results are annotated with their genomic location and gene annotation taken from the annotation files provided by Illumina. (*The coefficient value is equivalent to the magnitude of change in DNA methylation beta value and the direction of effect is in comparison to the control group)

Illumina Probe ID	*F* statistic	ANOVA *P* value	ANOVA *q* values	CHR	MAPINFO (hg19)	Gene name	ctrl v 17q12del			ctrl vs HNF1B mtn		
							*T* statistic	Coefficient*	*P* value	*T* statistic	Coefficient*	*P* value
cg06475972	47.017	4.00E-12	1.55E−06	17	34842357	ZNHIT3	9.045	0.043	5.06E−12	0.074	0.000	0.942
cg17953633	43.696	1.28E−11	2.49E−06	17	34957751	MRM1	8.532	0.069	2.97E−11	−0.417	− 0.003	0.678
cg24685795	40.277	4.52E−11	5.84E−06	17	34851276	ZNHIT3	− 8.075	− 0.107	1.47E−10	0.674	0.008	0.504
cg18269801	37.212	1.48E−10	1.44E−05	17	35732756	ACACA	− 8.088	− 0.061	1.40E−10	− 0.184	− 0.001	0.855
cg05527869	35.891	2.52E−10	1.95E−05	17	35294476	LHX1	7.988	0.025	1.99E−10	0.310	0.001	0.758
cg04264908	35.376	3.10E−10	2.01E−05	17	35303330		8.057	0.060	1.56E−10	0.716	0.005	0.477
cg22359664	32.781	9.19E−10	5.10E−05	17	35289432		7.094	0.044	4.75E−09	− 1.023	− 0.006	0.311
cg07037057	30.723	2.25E−09	0.000107005	17	35969138	SYNRG	7.684	0.076	5.84E−10	1.382	0.012	0.173
cg18103097	30.332	2.68E−09	0.000107005	17	35414317		− 7.386	− 0.071	1.68E−09	− 0.415	− 0.004	0.680
cg20151045	30.270	2.76E−09	0.000107005	17	35414114	AATF	− 7.242	− 0.051	2.80E−09	− 0.018	0.000	0.986
cg20951949	29.512	3.88E−09	0.000136867	17	34898162		− 7.047	− 0.078	5.60E−09	0.254	0.003	0.801
ch.17.958355F	28.600	5.89E−09	0.000190431	17	35991457	DDX52	7.365	0.055	1.81E−09	1.110	0.008	0.273
cg19678067	27.750	8.74E−09	0.00026103	17	34901173	GGNBP2	6.725	0.033	1.77E−08	− 0.509	− 0.002	0.613
cg06655187	27.194	1.14E−08	0.000315011	17	34842929	ZNHIT3	7.335	0.018	2.01E−09	1.984	0.004	0.053
cg03146993	26.915	1.30E−08	0.000335768	17	35297146	LHX1	6.987	0.041	6.95E−09	0.487	0.003	0.628
cg18574725	26.761	1.40E−08	0.000338748	17	34900600	GGNBP2	6.659	0.032	2.24E−08	− 0.370	− 0.002	0.713
cg06397845	26.545	1.55E−08	0.000353603	17	35732737	ACACA	− 6.809	− 0.066	1.31E−08	− 0.092	− 0.001	0.927
cg00356511	26.177	1.85E−08	0.000398715	17	35716273	ACACA	6.279	0.024	8.68E−08	− 1.035	− 0.004	0.306
cg02787306	25.017	3.26E−08	0.000665877	17	34957963	MRM1	6.652	0.044	2.29E−08	0.209	0.001	0.835
cg27614319	24.275	4.72E−08	0.000916069	17	35079061		6.453	0.040	4.68E−08	− 0.073	0.000	0.942
cg04993975	24.159	5.00E−08	0.000924571	17	35294472	LHX1	6.648	0.024	2.33E−08	0.555	0.002	0.582

**Fig. 4:**
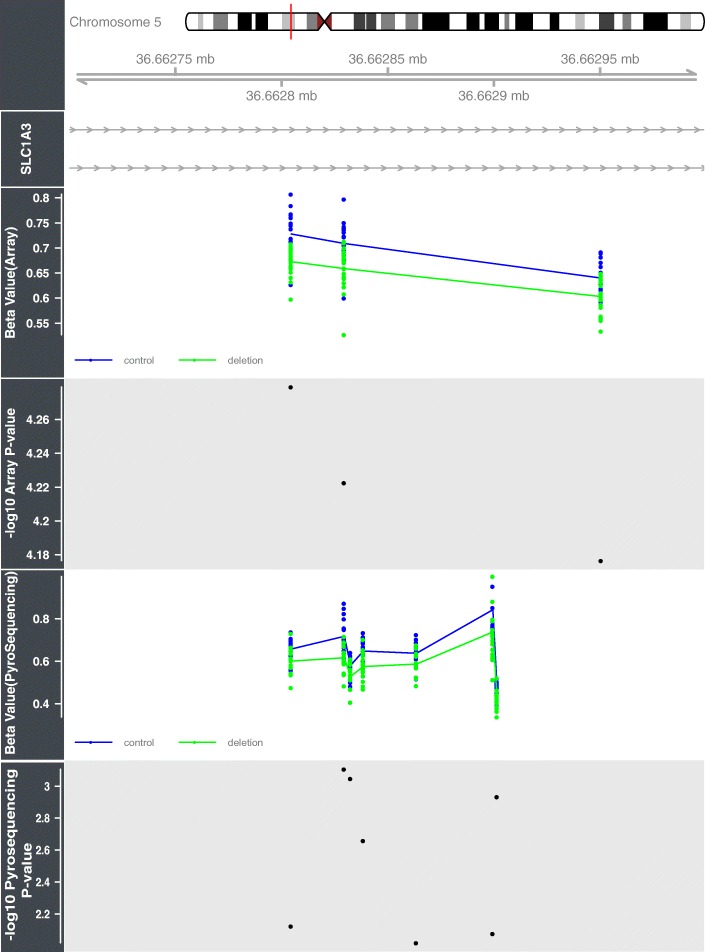
This figure shows the validation of the DMR located in the gene *SLC1A3* with bisulfite pyrosequencing. All CpGs assayed, including those not present on the 450K array, show significant hypomethylation in subjects harbouring a 17q12 deletion

DMR analysis comparing controls with the 17q12 deletion group identified a number of regions that were significant after correction for multiple comparisons using the comb-p program (Table 3). The majority of the DMRs identified were located in the 17q12 deletion region (Fig. 2). However, there were a few regions outside the deletion region that exhibited significant differential methylation; of interest were changes in the gene *SLC1A3* (*corrected P =* 4.71E−07, mean DNA methylation beta value Δ − 0.06), which has previously been implicated in Autism [40]. The DMR located in intron 3 of this gene was selected for verification with pyrosequencing. The assay designed only covered two of the three original probes comprising the DMR but did include an additional five CpG sites that were not present on the 450K array. The pyrosequencing data was converted into beta values (by dividing the pyrosequencing % values by 100) and regressed against *HNF1B* status with covariates for age, diabetes status, gender and cell composition as in the initial analysis. The five additional sites assayed by the pyrosequencer all showed significant hypomethylation (*P* < 0.01) in the 17q12 deletion patients compared to the controls (see Fig. 4).

**Table 3:**
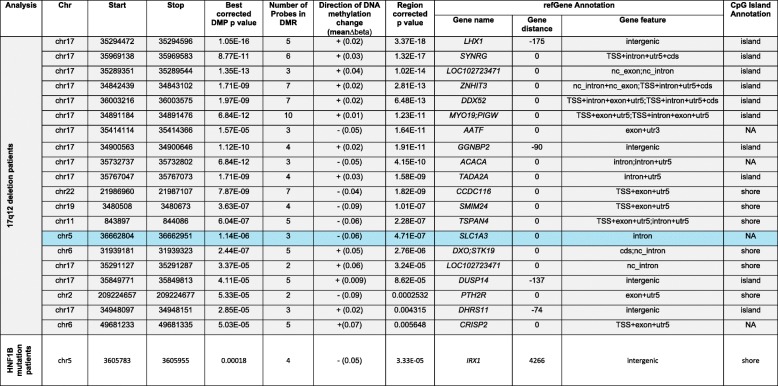
Differential methylation regional (DMR) analysis on the two HNF1B deficient groups of patients compared to the controls (highlighted in blue is the DMR residing in the gene *SLC1A3* which was validated using pyrosequencing)

In contrast, only one significant DMR was located in the analysis comparing controls with the *HNF1B* mutation group (see Table 3).

## Discussion

In this study, we have assessed genome-wide patterns of DNA methylation in DNA derived from the blood of individuals with HNF1B-associated disease along with matched healthy controls. The aim of this experiment was to determine if there was an epigenetic signature that can distinguish *HNF1B* intragenic mutation patients from those with a 17q12 deletion. While both genotype groups result in developmental kidney disease only individuals with the 17q12 deletion appear to have a greater risk of developing neuropsychiatric symptoms, suggesting the contribution of other genes or regulatory mechanisms are at play.

We first examined site-specific genome-wide patterns of DNA methylation in the three groups studied (*HNF1B* mutations, 17q12 deletion and controls) and found striking changes in DNA methylation of the 17q12 deletion region. The 21 DMPs that reached our experimental-wide significance threshold all mapped to the 17q12 deletion region. While we do see a drop in signal intensity in this region in the deletion samples, it is consistent across the methylated and unmethylated channels so will not influence the calculated beta values. Interestingly, the direction of effect is not consistent, with probes exhibiting both gains and losses of DNA methylation suggesting the observed changes are not a technical artefact on the array resulting from the haploinsufficiency of the region. Comparing the two *HNF1B* genotype groups with controls separately confirmed that the association located to the 1.3 Mb deletion at chromosome 17q12 was driven by the 17q12 deletion samples.

Given that DNA methylation at adjacent probes is often correlated, we employed regional-based analysis and identified 20 DMRs in the 17q12 deletion samples, 13 of which were located in the 1.3 Mb deletion region. The most significant DMR mapped upstream of the gene *LHX1 (*corrected *P =* 3.37E−18, mean DNA methylation beta Δ + 0.02) (see Additional file 5: Figure S2), which is known to play a role in brain development and function [41, 42] as well as being directly regulated by *HNF1B* during kidney development [43, 44].

These observations suggest a possible compensatory mechanism mediated by DNA methylation reacting to haploinsufficiency in the vicinity of the deletion. The enrichment of DMRs in this region indicates that this phenomenon is not entirely random and highlights a number of loci in the region that may have regulatory importance. Recently, DNA methylation has been found to have a major role in “fine-tuning” the expression of genes located in CNVs found in various different cancers [45].

While there is a substantial enrichment of DMRs in the deletion region a number of DMRs were identified elsewhere in the genome, one of which was located in the gene *SLC1A3* on chromosome 5. Genetic variations in this gene have been linked to autism and expression changes in this gene have been identified in schizophrenia patients; both these disorders have been associated with the 17q12 deletion [18, 46]. Further work is needed to identify whether alterations in the DNA methylation profile in this gene affect gene function.

So far, we have concentrated on the differences in the DNA methylation signature in the 17q12 deletion subjects which are not shared by the *HNF1B* mutation subjects. However, these two groups have similar phenotypes and this was reflected in the DNA methylation profile of the two groups once the probes located in the 17q12 region are removed, indicating that there is a shared network of genes that are dysregulated due to the haploinsufficency of *HNF1B* (see Fig. 3 and Additional file 4: Table S3). The majority of these shared probes are significantly hypomethylated in both patient groups compared to controls and include cg01445838 (17q12del vs control *P* = 5.38E−05, coefficient = − 0.064, HNF1B mutation vs control *P* = 0.000213, coefficient = − 0.052), which is located in the maternally imprinted gene *PLAGL1*. This gene is known to be involved in fetal growth and is necessary for normal pancreatic islet development [47]. Furthermore, loss of DNA methylation at this locus results in transient neonatal diabetes mellitus which is postulated to be caused by an increase in expression of PLAGL1 [48].

The main limitation of this study is that it uses blood-derived DNA and not disease-relevant tissue and/or cells (e.g. the kidney or the brain). However, genetic-mediated changes in DNA methylation (methylation QTLS) have been found to be considerably stable across different tissues and cell types [39] suggesting that we may be able to extrapolate these findings to other more relevant tissues.

Clinical details were taken from information available at the time of referral for genetic testing; there may be patient factors present, including medication history, which we have been unable to control for and may have influenced our results. Further, we also do not have detailed psychiatric data available for the samples included in this study so we cannot make any assumptions regarding the DNA methylation status of the 17q12 deletion and the increased prevalence of psychiatric symptoms. Many of the individuals were too young to be assessed for schooling difficulties and the features of neurodevelopmental disease often do not become apparent until children are older.

## Conclusion

We have identified significant DNA methylation alterations in individuals with a 17q12 heterozygous deletion, which localise to a 1.3 Mb deletion region. The observed changes in DNA methylation at this locus are not randomly dispersed and occur in clusters suggesting a regulatory mechanism reacting to haploinsufficiency across the deleted region. Along with these deletion-specific changes in DNA methylation, we also identified a common DNA methylation signature in both genotype groups, indicating that haploinsufficiency of *HNF1B* impacts on the methylome of a number of genes. Further work should investigate the role of these genes in the manifestation of the various phenotypes associated with deficiency of HNF1B and investigate gene expression changes associated with DNA methylation status across the deletion region in disease relevant tissues. Also the increased prevalence of psychiatric symptoms in patients with the 17q12 deletion should be considered and whether alterations in DNA methylation in this region differ depending on psychiatric diagnosis.

Due to the rare nature of HNF1B mutations/deletions, validation of these findings in an independent population was not possible in this study but further work should investigate DNA methylation changes *in cis* of other more common disease associated copy number variations.

To conclude, we have identified several genes that are differentially methylated in HNF1B-associated disease, some of which are specific to 17q12 deletion subjects. Further, this study is, as far as we are aware, the first to document organised changes in DNA methylation across a large deletion region suggestive of a compensational role of this epigenetic modification.

## Additional files

Additional file 1: **Table S1.** SLC1A3 bisulfite pyrosequencing assay conditions. (XLSX 11 kb)

Additional file 2: **Figure S1.** This figure illustrates the extent of the 17q12 deletion in each patient as estimated by the CNV calling algorithm within the CHAMP package. (PDF 15 kb)

Additional file 3: **Table S2.** All anlaysed probes from the ANOVA test with a *p*-value <0.01. (XLSX 688 kb)

Additional file 4: **Table S3.** Probes that are significantly differentaly methylated in both of the separate group analyses (note that all probes are in the same direction in both analyses). (XLSX 19 kb)

Additional file 5: **Figure S2.** This Figure shows four significant differentially methylated regions (DMRs) identified between controls and 17q12 deletion carriers. A) *SYNRG* (corrected *P* = 1.32E-17), B) *AATF* (corrected *P* = 1.64E-11). C) *LHX1* (corrected *P =* 3.37E-18), D) *SMIM24* (corrected *P =* 1.01E-07). (PDF 223 kb)

## Abbreviations

ANOVA: Analysis of variance
CNV: Copy number variant
DMP: Differentially methylated position
DMR: Differentially methylated region
HNF1B: Hepatocyte nuclear factor 1β
SNP: Single nucleotide polymorphism
