# Supplementary figures and images for: Genome-wide methylomic analysis in individuals with HNF1B intragenic mutation and 17q12 microdeletion
Source: Clin Epigenetics. 2018 Jul 18;10:97. doi: 10.1186/s13148-018-0530-z (PMC6052548; doi:10.1186/s13148-018-0530-z)

Chromosome 17

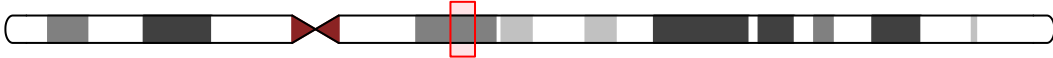

35 mb

36 mb

35.5 mb

ENSEMBL

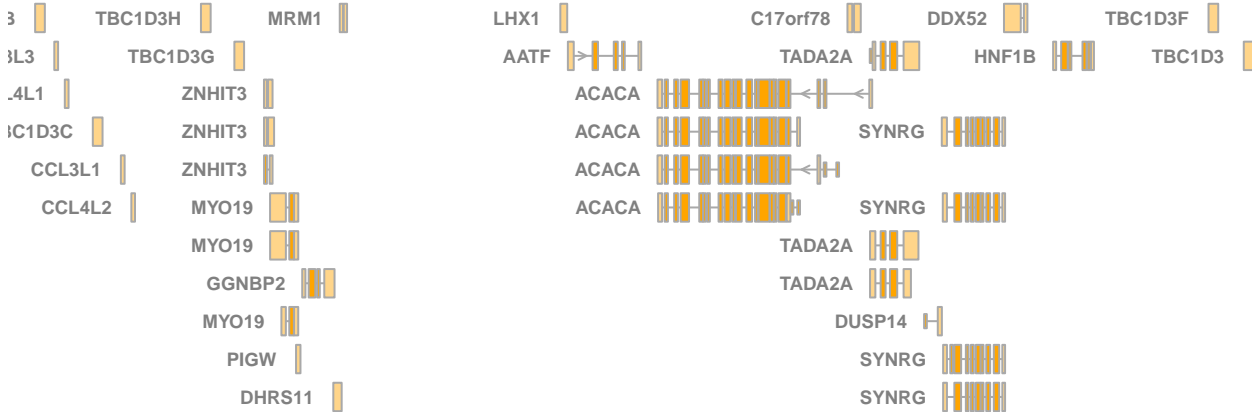

Estimated Patient Deletions

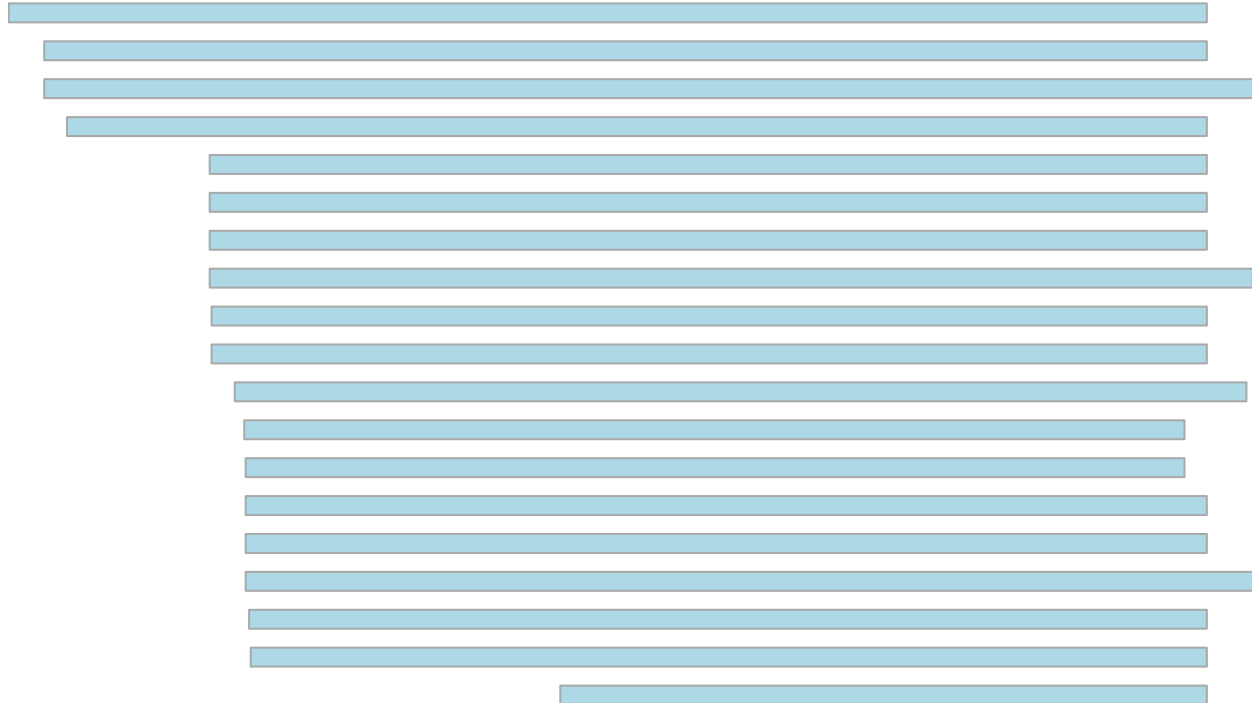

Supplement: Supplementary file 2 — Figure S1. This figure illustrates the extent of the 17q12 deletion in each patient as estimated by the CNV calling algorithm within the CHAMP package. (PDF 15 kb) [file 13148_2018_530_MOESM2_ESM.pdf]

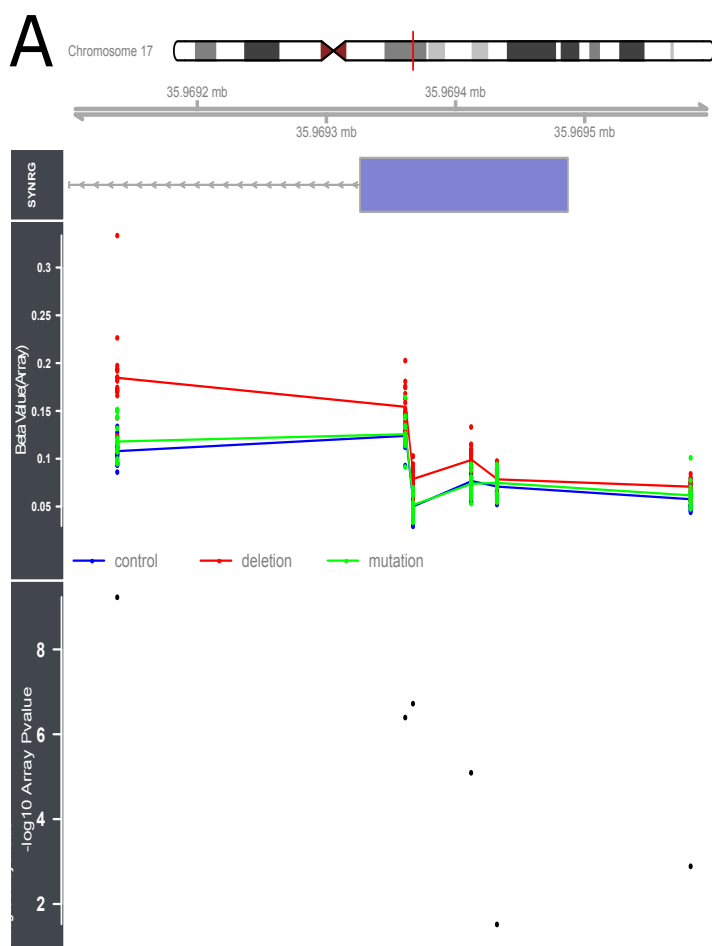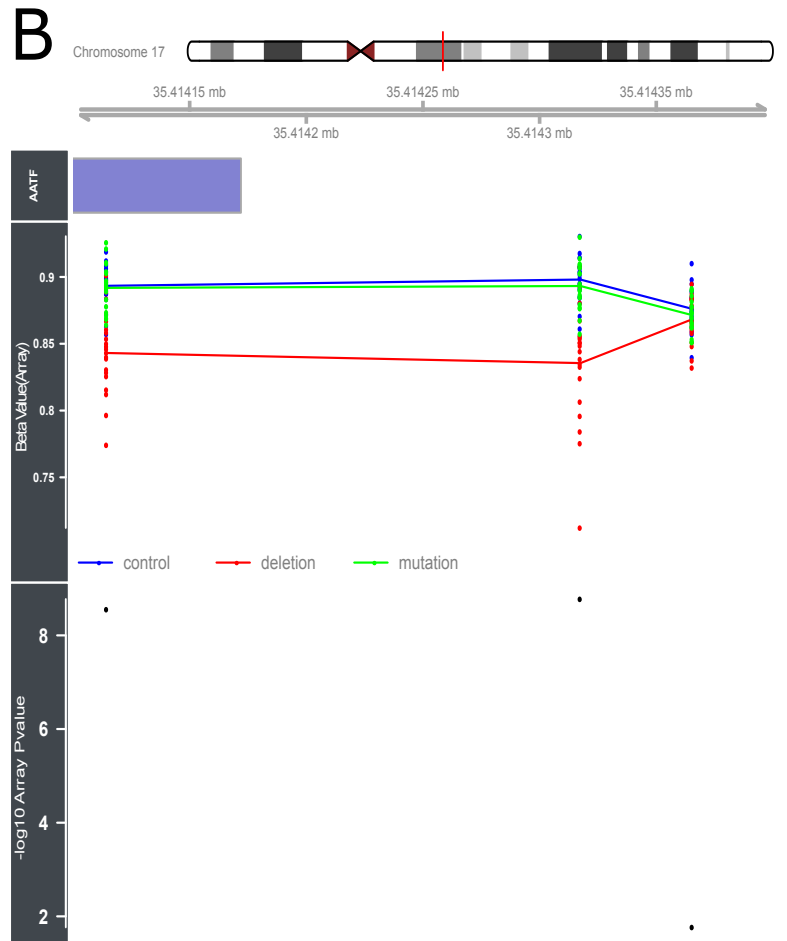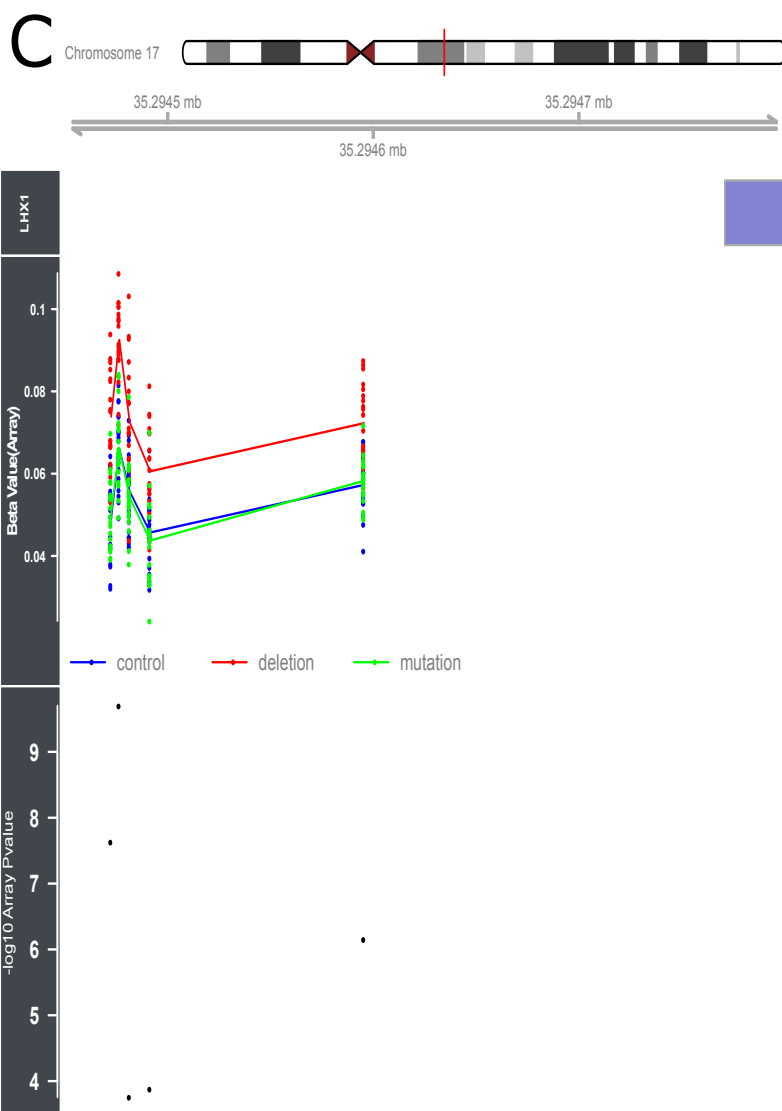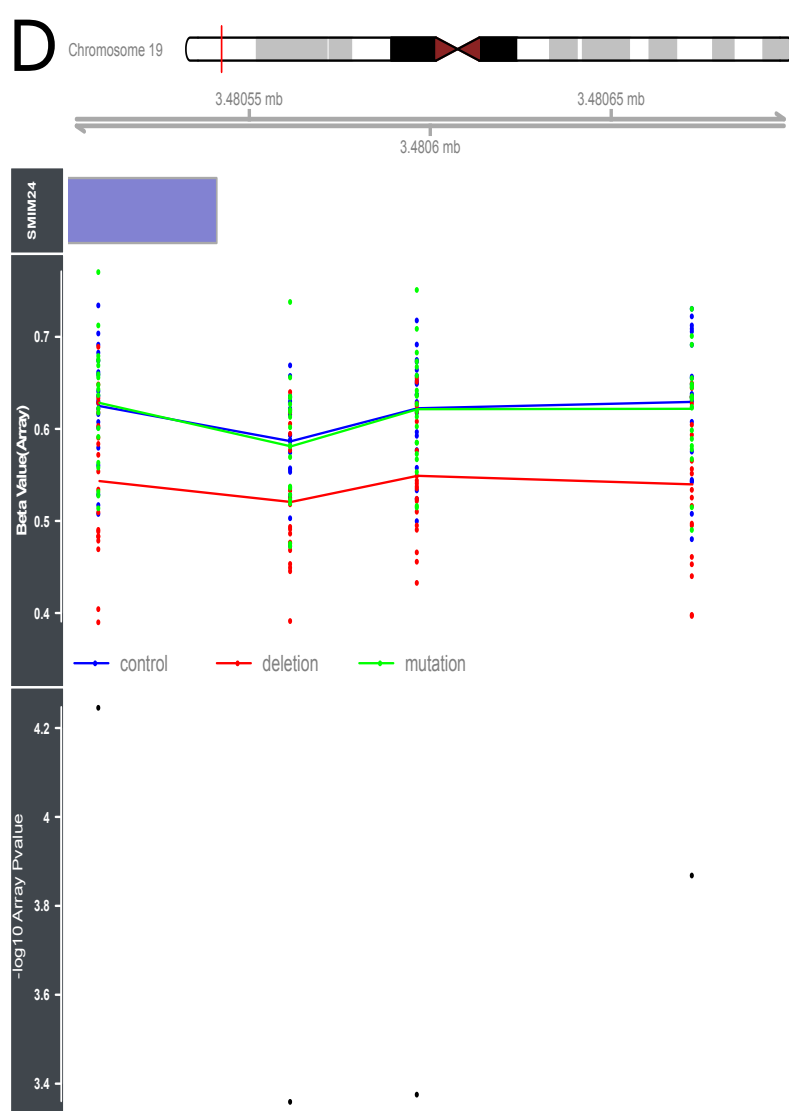

Supplement: Supplementary file 5 — Figure S2. This Figure shows four significant differentially methylated regions (DMRs) identified between controls and 17q12 deletion carriers. A) SYNRG (corrected P = 1.32E-17), B) AATF (corrected P = 1.64E-11). C) LHX1 (corrected P = 3.37E-18), D) SMIM24 (corrected P = 1.01E-07). (PDF 223 kb) [file 13148_2018_530_MOESM5_ESM.pdf]
